# Remembrance programs in pediatric care: Transforming grief into community resilience

**DOI:** 10.1017/S1478951525100679

**Published:** 2025-08-13

**Authors:** Rikas Saputra, Raja Rahima Munawarah Raja Ahmad, Isnaria Rizki Hayati, Neni Noviza, Yenni Lidyawati

**Affiliations:** 1 Fakultas Dakwah dan Komunikasi, Universitas Islam Negeri Raden Fatah Palembang, Palembang, Indonesia; 2 Guidance and Counseling Islamic, Universitas Islam Negeri Sultan Syarif Kasim, Pekanbaru, Indonesia; 3 Guidance and Counseling, Universitas Riau, Pekanbaru, Indonesia; 4 Department Guidance and Counseling, Universitas Islam Negeri Raden Fatah Palembang, Palembang, Indonesia; 5 Department Pendidikan dan Sastra Indonesia, Fakultas Keguruan dan Ilmu Pendidikan Universitas Sriwijaya, Palembang, Indonesia

Dear Editor,

We greatly appreciate the article by Wiener et al. 2024 entitled “Making space for grief: The impact of remembrance programmes for paediatric healthcare providers,” published in Palliative and Supportive Care. The authors offer a timely and compelling exploration of how structured remembrance programs such as the Pediatric Remembrance Ceremony (PRC) and Good Grief and Chocolate at Noon (GGCN) can foster healing, reflection, and solidarity among pediatric healthcare staff facing the profound emotional burden of patient loss.

Wiener et al. aptly highlight an often-overlooked yet critical aspect of pediatric palliative care: the emotional well-being of healthcare providers. The study demonstrates that remembrance ceremonies offer more than mere rituals; they create a safe space for staff to remember and process grief collectively. Interestingly, the authors show that the shift to virtual and hybrid formats during the COVID-19 pandemic expanded access and inclusivity, marking a new evolution in institutional grief support in the digital age.

The findings of this article align with the growing consensus that pediatric healthcare workers’ emotional burden requires a planned institutional response (Costa et al, 2025). Personal rituals such as lighting candles, playing music, and sharing stories validate individual experiences of loss and strengthen community resilience, as evidenced by the fact that these practices can reduce compassion fatigue and support professional sustainability (Danbolt and Stifoss-Hanssen 2017; O’Callaghan et al 2018).

However, several questions arise. First, this study was conducted at a single research institution, so the potential for generalization remains limited. Comparative studies across various healthcare environments and cultures are needed to understand how remembrance practices can be adapted or developed collaboratively in different contexts (Haraldseid-Driftland et al 2022). Second, while subjective benefits have been demonstrated, future research should incorporate longitudinal data or standardized psychological instruments (e.g., burnout, secondary traumatic stress) to measure long-term impacts objectively (Moreno-Jiménez et al 2023).

It is also important to note that staff desires flexibility in virtual, in-person, or hybrid formats, emphasizing the importance of inclusivity in grief support (Cherniwchan 2022). Expanding these options can ensure more equitable access, particularly for dispersed or work remotely, and could become a new standard for institutional grief support post-pandemic.

In conclusion, this article is important to the literature on pediatric palliative care and staff well-being. By prioritizing structured remembrance programs, healthcare institutions can transform spaces of silent suffering into meaningful, connected, and hopeful communities. We encourage further investment and research into adaptive, evidence-based grief support models so that patients and their carers receive equal respect.
